# Supplementary material for: MicroRNA‐34/449 controls mitotic spindle orientation during mammalian cortex development
Source: EMBO J. 2016 Oct 5;35(22):2386–98. doi: 10.15252/embj.201694056 (PMC5109238; doi:10.15252/embj.201694056)
Supplement: Supplementary file 1 — Appendix [file EMBJ-35-2386-s001.docx]

**Appendix**

**Table of Contents**

**Appendix Table S1………………………….2-5**

**Appendix Table S2………………………….6-15**

**Appendix Supplementary Methods………..16-22**

**Appendix References………………………..23**

**Appendix Table S1 - miRNA mimic ranking (mitotic duration screening)**

| **microRNA mimic** | **mean Z score (mitotic timing)** |
| --- | --- |
| hsa-miR-433 | -0.686939221 |
| hsa-miR-335 | -0.63148327 |
| hsa-miR-101 | -0.626079009 |
| hsa-miR-219-5p | -0.616197838 |
| hsa-miR-125b-2* | -0.591006247 |
| hsa-miR-151-3p | -0.471429155 |
| hsa-miR-30c-1* | -0.435207424 |
| hsa-miR-125b-1* | -0.426525429 |
| hsa-miR-409-3p | -0.392419559 |
| hsa-miR-146b-5p | -0.385585029 |
| hsa-miR-487b | -0.382374562 |
| hsa-miR-143 | -0.37664955 |
| hsa-miR-320c | -0.334701335 |
| hsa-miR-26a | -0.329183471 |
| hsa-miR-125b | -0.318725645 |
| hsa-miR-146a | -0.318162226 |
| hsa-miR-448 | -0.318081169 |
| hsa-miR-342-3p | -0.316004342 |
| hsa-miR-152 | -0.311850687 |
| hsa-miR-374a | -0.311006538 |
| hsa-miR-199a-3p | -0.305236615 |
| hsa-miR-25 | -0.30428531 |
| hsa-miR-485-5p | -0.304244902 |
| hsa-miR-125a-3p | -0.301924513 |
| hsa-miR-708 | -0.301804658 |
| hsa-miR-99b | -0.300676653 |
| hsa-miR-423-3p | -0.30034106 |
| hsa-miR-378 | -0.299460298 |
| hsa-let-7a | -0.292776063 |
| hsa-miR-23b | -0.289010329 |
| hsa-miR-221 | -0.285217439 |
| hsa-miR-107 | -0.28366549 |
| hsa-miR-296-3p | -0.278176825 |
| hsa-miR-320b | -0.223419671 |
| hsa-miR-30c-2* | -0.174119814 |
| hsa-miR-345 | -0.171619735 |
| hsa-miR-34b | -0.169096204 |
| hsa-miR-9* | -0.164015145 |
| hsa-miR-99a* | -0.16139415 |
| hsa-miR-192 | -0.15669885 |
| hsa-miR-598 | -0.153036963 |
| hsa-miR-33b | -0.148801398 |
| hsa-miR-106b* | -0.128879384 |
| hsa-miR-30a* | -0.106322885 |
| hsa-miR-30e* | -0.105213021 |
| hsa-miR-320d | -0.06731035 |
| hsa-miR-124 | -0.039589068 |
| hsa-miR-151-5p | -0.03332901 |
| hsa-miR-33a | -0.028578863 |
| hsa-miR-186 | -0.027074857 |
| hsa-miR-543 | -0.024960969 |
| hsa-miR-222 | -0.016576946 |
| hsa-miR-331-3p | -0.016001643 |
| hsa-miR-320a | -0.010351345 |
| hsa-miR-494 | -0.008261599 |
| hsa-miR-379 | -0.00742364 |
| hsa-miR-30b | -0.007416272 |
| hsa-miR-30d | -0.004256818 |
| hsa-miR-26b | -0.002172621 |
| hsa-miR-21 | -7.37E-06 |
| hsa-let-7e | 0.002069459 |
| hsa-miR-363 | 0.005731114 |
| hsa-miR-92b | 0.006897065 |
| hsa-miR-9 | 0.012608341 |
| hsa-miR-98 | 0.013676229 |
| hsa-miR-542-3p | 0.013690967 |
| hsa-miR-185 | 0.018956723 |
| hsa-miR-99a | 0.026262584 |
| hsa-miR-451 | 0.026406409 |
| hsa-miR-7 | 0.03264769 |
| hsa-miR-499-5p | 0.033693473 |
| hsa-miR-374b | 0.035864665 |
| hsa-miR-191 | 0.042473801 |
| hsa-miR-495 | 0.043738098 |
| hsa-let-7f | 0.044283936 |
| hsa-miR-140-3p | 0.048666528 |
| hsa-miR-323-3p | 0.049098005 |
| hsa-miR-181b | 0.050026708 |
| hsa-miR-369-5p | 0.055035954 |
| hsa-miR-30a | 0.060589359 |
| hsa-miR-298 | 0.184975639 |
| hsa-miR-219-1-3p | 0.189951835 |
| hsa-miR-130b | 0.19597287 |
| hsa-miR-25* | 0.205050298 |
| hsa-miR-301b | 0.224849675 |
| hsa-miR-487a | 0.255534254 |
| hsa-miR-106a | 0.277335095 |
| hsa-miR-30c | 0.309561327 |
| hsa-miR-24 | 0.310666059 |
| hsa-miR-125a-5p | 0.311169367 |
| hsa-miR-206 | 0.312245786 |
| hsa-miR-134 | 0.316005503 |
| hsa-miR-15a | 0.316810643 |
| hsa-miR-30e | 0.31803075 |
| hsa-miR-29a | 0.318523375 |
| hsa-miR-130a | 0.321883419 |
| hsa-miR-503 | 0.328176436 |
| hsa-miR-17 | 0.347518648 |
| hsa-miR-340 | 0.347585305 |
| hsa-miR-382 | 0.348111758 |
| hsa-miR-93 | 0.35344153 |
| hsa-miR-29b | 0.354768836 |
| hsa-miR-181c | 0.386055733 |
| hsa-miR-92a | 0.395556766 |
| hsa-miR-20a | 0.398009708 |
| hsa-miR-23a | 0.403122372 |
| hsa-miR-100 | 0.404187367 |
| hsa-miR-29c | 0.406089976 |
| hsa-miR-20b | 0.522028868 |
| hsa-miR-27b | 0.625727781 |
| hsa-let-7c | 0.629867861 |
| hsa-miR-34a | 0.630898906 |
| hsa-let-7b | 0.635052561 |
| hsa-miR-27a | 0.640353999 |
| hsa-let-7g | 0.669715304 |
| hsa-let-7d | 0.670387616 |
| hsa-miR-181d | 0.733382614 |
| hsa-miR-106b | 0.771419733 |
| hsa-miR-127-3p | 0.961581047 |
| hsa-miR-128 | 0.972642239 |
| hsa-let-7i | 0.991201921 |
| hsa-miR-34c-5p | 0.994629092 |
| hsa-miR-532-5p | 1.014239793 |
| hsa-miR-219-2-3p | 1.034100742 |
| hsa-miR-181a | 1.097398761 |
| hsa-miR-541 | 1.118080452 |
| hsa-miR-106a* | 1.128853668 |
| hsa-miR-30b* | 1.398246699 |
| hsa-miR-15b* | 1.862626278 |
| hsa-miR-449a | 1.946499742 |
| hsa-miR-30d* | 2.240702265 |
| hsa-miR-135b* | 2.273586098 |
| hsa-miR-99b* | 2.314015891 |
| hsa-miR-135a* | 2.578627864 |
| hsa-miR-449b | 3.153400308 |

Note:* represents miRNA product from the 3’arm of the hairpin

**Appendix Table S2 - Downregulated genes in HeLa cells under miR-449 mimic overexpression**

| **Accession** | **Entrez Gene ID** | **Gene Symbol** |
| --- | --- | --- |
| NM_003733 | 8638 | OASL |
| NM_000543 | 6609 | SMPD1 |
| NM_021627 | 59340 | SENP2 |
| NM_078467 | 1026 | CDKN1A |
| XM_001127426 | 728400 | LOC728416 |
| NM_004864 | 9518 | GDF15 |
| NM_001547 | 3433 | IFIT2 |
| XR_015964 | 729300 | LOC729255 |
| NM_002166 | 3398 | ID2 |
| NM_002201 | 3669 | ISG20 |
| ENST00000374274 | 29930 | SNX12 |
| NM_006417 | 10560 | IFI44 |
| NM_001549 | 3437 | IFIT3 |
| NM_032121 | 84060 | MAGT1 |
| NM_005101 | 9636 | ISG15 |
| NM_002166 | 3398 | ID2 |
| NM_016202 | 51160 | ZNF580 |
| NM_001078173 | 441500 | FAM127C |
| NM_019593 | 56260 | RP5-1022P6.2 |
| NM_178457 | 128600 | ZNF831 |
| NM_001547 | 3433 | IFIT2 |
| NM_015933 | 51370 | CCDC72 |
| NM_005211 | 1436 | CSF1R |
| NM_213636 | 9260 | PDLIM7 |
| NM_007197 | 11210 | FZD10 |
| NM_014454 | 27240 | SESN1 |
| NM_006270 | 6237 | RRAS |
| NM_006186 | 4929 | NR4A2 |
| NM_001039590 | 8239 | USP9X |
| NM_016256 | 51170 | NAGPA |
| NM_053284 | 117200 | WFIKKN1 |
| NM_015087 | 23110 | SPG20 |
| NM_017842 | 55650 | SLC48A1 |
| NM_198549 | 375000 | FAM73A |
| NM_194328 | 152000 | RNF38 |
| NM_006408 | 10550 | AGR2 |
| NM_005269 | 2735 | GLI1 |
| NM_032683 | 84770 | MPV17L2 |
| NM_002158 | 3344 | FOXN2 |
| NM_001083112 | 2820 | GPD2 |
| NM_006353 | 10470 | HMGN4 |
| NM_000422 | 3872 | KRT17 |
| NM_020428 | 57150 | SLC44A2 |
| NM_138392 | 92800 | SHKBP1 |
| NM_006186 | 4929 | NR4A2 |
| NM_053279 | 83650 | FAM167A |
| NM_017582 | 55580 | UBE2Q1 |
| NM_020428 | 57150 | SLC44A2 |
| NM_199001 | 375800 | C9orf169 |
| NM_000245 | 4233 | MET |
| NM_000434 | 4758 | NEU1 |
| NM_024667 | 79720 | VPS37B |
| NM_002985 | 6352 | CCL5 |
| NM_000526 | 3861 | KRT14 |
| NM_024923 | 23220 | NUP210 |
| NM_199001 | 375800 | C9orf169 |
| NM_001025252 | 7163 | TPD52 |
| NM_016626 | 51320 | MEX3C |
| NM_014314 | 23590 | DDX58 |
| NM_018490 | 55370 | LGR4 |
| NM_004308 | 392 | ARHGAP1 |
| NM_001632 | 250 | ALPP |
| NM_020375 | 57100 | C12orf5 |
| NM_005860 | 10270 | FSTL3 |
| NM_007329 | 1755 | DMBT1 |
| NM_001113434 | 339300 | C17orf51 |
| NM_032470 | 7148 | TNXB |
| NM_000945 | 5534 | PPP3R1 |
| NM_001312 | 1397 | CRIP2 |
| NM_016623 | 51570 | FAM49B |
| NM_003004 | 6398 | SECTM1 |
| NM_003059 | 6583 | SLC22A4 |
| NM_030791 | 81540 | SGPP1 |
| NM_004665 | 8875 | VNN2 |
| NM_007362 | 22920 | NCBP2 |
| NM_017775 | 54900 | TTC19 |
| NM_017768 | 55630 | LRRC40 |
| NM_030579 | 80780 | CYB5B |
| NM_025146 | 80220 | NAT13 |
| NM_001008392 | 10220 | CTDSPL |
| NM_152737 | 221700 | RNF182 |
| NM_152424 | 139300 | FAM123B |
| NM_080670 | 113800 | SLC35A4 |
| XM_001133504 | 729200 | LOC729242 |
| NM_003877 | 8835 | SOCS2 |
| NM_002158 | 3344 | FOXN2 |
| NM_198282 | 340100 | TMEM173 |
| NM_000075 | 1019 | CDK4 |
| NM_020647 | 56700 | JPH1 |
| NM_024111 | 79090 | CHAC1 |
| NM_001543 | 3340 | NDST1 |
| NM_030805 | 81560 | LMAN2L |
| NM_002964 | 6279 | S100A8 |
| NM_012262 | 9653 | HS2ST1 |
| NM_002906 | 5962 | RDX |
| NM_017762 | 54890 | MTMR10 |
| NR_027425 | 1E+08 | FAM66D |
| NM_032899 | 84980 | FAM83A |
| NM_198474 | 283300 | OLFML1 |
| NM_001127663 | 2934 | GSN |
| NR_026861 | 90630 | C6orf176 |
| NM_018269 | 55260 | ADI1 |
| NM_002073 | 2781 | GNAZ |
| NM_001004356 | 53830 | FGFRL1 |
| NM_005491 | 10050 | MAMLD1 |
| NM_019008 | 54470 | SMCR7L |
| NM_152739 | 3205 | HOXA9 |
| NM_014631 | 9644 | SH3PXD2A |
| NM_175866 | 127900 | UHMK1 |
| NM_004585 | 5920 | RARRES3 |
| NM_016417 | 51220 | GLRX5 |
| XR_078607 | 1E+08 | LOC100289026 |
| NM_020412 | 57130 | CHMP1B |
| NM_005318 | 3005 | H1F0 |
| NM_024119 | 79130 | DHX58 |
| NM_145284 | 159100 | FAM122B |
| NM_006148 | 3927 | LASP1 |
| NM_003779 | 8703 | B4GALT3 |
| NM_004055 | 726 | CAPN5 |
| NM_024625 | 56830 | ZC3HAV1 |
| NR_029392 | 400600 | LOC400578 |
| NM_003270 | 7105 | TSPAN6 |
| NM_020342 | 57180 | SLC39A10 |
| NM_014774 | 9813 | KIAA0494 |
| NR_003226 | 172 | AFG3L1 |
| XM_002347413 | 1E+08 | LOC100128430 |
| NM_080391 | 8073 | PTP4A2 |
| NM_012166 | 26270 | FBXO10 |
| NM_017842 | 55650 | SLC48A1 |
| NM_004060 | 900 | CCNG1 |
| NM_004661 | 8697 | CDC23 |
| NM_001040455 | 51090 | SIDT2 |
| NM_012392 | 553100 | PEF1 |
| NM_019008 | 54470 | SMCR7L |
| NM_001142590 | 4802 | NFYC |
| NM_006294 | 7381 | UQCRB |
| NM_182908 | 10200 | DHRS2 |
| NM_014759 | 9796 | PHYHIP |
| NM_012238 | 23410 | SIRT1 |
| NM_080657 | 91540 | RSAD2 |
| NM_145166 | 93000 | ZBTB47 |
| NM_001030001 | 6235 | RPS29 |
| NM_018201 | 54660 | TBC1D13 |
| NM_004364 | 1050 | CEBPA |
| NM_000088 | 1277 | COL1A1 |
| NM_000433 | 4688 | NCF2 |
| NM_138287 | 151600 | DTX3L |
| NM_001040167 | 3955 | LFNG |
| NM_000107 | 1643 | DDB2 |
| NM_024111 | 79090 | CHAC1 |
| NM_032575 | 84660 | GLIS2 |
| NM_012320 | 23660 | PLA2G15 |
| NM_015679 | 27000 | TRUB2 |
| NM_001008709 | 5499 | PPP1CA |
| NM_003908 | 8894 | EIF2S2 |
| NM_052965 | 116500 | TSEN15 |
| NM_001654 | 369 | ARAF |
| NM_001703 | 576 | BAI2 |
| NM_032121 | 84060 | MAGT1 |
| NM_022745 | 64760 | ATPAF1 |
| NM_004096 | 1979 | EIF4EBP2 |
| NM_004475 | 2319 | FLOT2 |
| NM_000966 | 5916 | RARG |
| NM_006829 | 10970 | C10orf116 |
| NM_033102 | 85410 | SLC45A3 |
| NM_018660 | 55890 | ZNF395 |
| NM_032486 | 84520 | DCTN5 |
| NM_024567 | 79620 | HMBOX1 |
| NM_015455 | 57470 | CNOT6 |
| NM_201649 | 6536 | SLC6A9 |
| NM_021133 | 6041 | RNASEL |
| NM_032173 | 84130 | ZNRF3 |
| NM_144563 | 22930 | RPIA |
| NM_147190 | 91010 | LASS5 |
| NM_004230 | 9294 | S1PR2 |
| NM_030792 | 81540 | GDPD5 |
| NM_005346 | 3304 | HSPA1B |
| NM_000270 | 4860 | NP |
| NM_016647 | 51340 | C8orf55 |
| NM_007221 | 11240 | PMF1 |
| NM_152371 | 127300 | C1orf93 |
| NM_002639 | 5268 | SERPINB5 |
| NM_001703 | 576 | BAI2 |
| NM_174907 | 152000 | PPP4R2 |
| NM_022147 | 64110 | RTP4 |
| NM_138444 | 115200 | KCTD12 |
| NM_024636 | 79690 | STEAP4 |
| NM_013354 | 29880 | CNOT7 |
| NM_004739 | 9219 | MTA2 |
| NM_203434 | 389800 | IER5L |
| NM_025263 | 80740 | PRR3 |
| NM_004060 | 900 | CCNG1 |
| NM_014400 | 27080 | LYPD3 |
| NM_001025091 | 23 | ABCF1 |
| NM_152930 | 8904 | CPNE1 |
| NM_005099 | 9507 | ADAMTS4 |
| NM_001956 | 1907 | EDN2 |
| NM_006838 | 10990 | METAP2 |
| NM_203347 | 389800 | LCN15 |
| NM_015093 | 23120 | MAP3K7IP2 |
| NM_001003799 | 445300 | TARP |
| NM_032121 | 84060 | MAGT1 |
| NM_014747 | 9783 | RIMS3 |
| NM_001099220 | 643600 | ZNF862 |
| NM_032799 | 84880 | ZDHHC12 |
| NM_054023 | 117200 | SCGB3A2 |
| NM_014971 | 22980 | EFR3B |
| NM_004451 | 2101 | ESRRA |
| NM_022912 | 65060 | REEP1 |
| NM_004599 | 6721 | SREBF2 |
| NM_006036 | 9581 | PREPL |
| NM_001280 | 1153 | CIRBP |
| NM_133639 | 171200 | RHOV |
| NM_004451 | 2101 | ESRRA |
| NM_005187 | 863 | CBFA2T3 |
| NM_053056 | 595 | CCND1 |
| NM_173216 | 6480 | ST6GAL1 |
| NM_181886 | 7323 | UBE2D3 |
| NM_138433 | 113700 | KLHDC7B |
| NM_001006605 | 388600 | FAM69A |
| NM_002193 | 3625 | INHBB |
| NM_018361 | 55330 | AGPAT5 |
| NM_201649 | 6536 | SLC6A9 |
| NM_198841 | 158300 | FAM120AOS |
| NM_001144070 | 8714 | ABCC3 |
| NM_023080 | 65260 | C8orf33 |
| NM_212479 | 10770 | ZMYND11 |
| NM_003779 | 8703 | B4GALT3 |
| NM_002292 | 3913 | LAMB2 |
| NM_005704 | 10080 | PTPRU |
| NM_005517 | 3151 | HMGN2 |
| NM_002081 | 2817 | GPC1 |
| NM_030927 | 81620 | TSPAN14 |
| NM_021959 | 6992 | PPP1R11 |
| NM_020150 | 56680 | SAR1A |
| NM_014856 | 9909 | DENND4B |
| NM_005371 | 4234 | METTL1 |
| NM_152310 | 83400 | ELOVL3 |
| NM_001135993 | 125500 | TTC39C |
| NM_014873 | 9926 | LPGAT1 |
| NM_017851 | 54960 | PARP16 |
| NM_080669 | 113200 | SLC46A1 |
| NM_021130 | 5478 | PPIA |
| NM_007198 | 11210 | PROSC |
| NM_000841 | 2914 | GRM4 |
| NM_144593 | 121300 | RHEBL1 |
| NM_021198 | 58190 | CTDSP1 |
| ENST00000373670 | 54540 | RC3H2 |
| NM_032885 | 84970 | ATG4D |
| NM_000161 | 2643 | GCH1 |
| NM_000210 | 3655 | ITGA6 |
| NM_016407 | 51510 | C20orf43 |
| NM_007176 | 11160 | C14orf1 |
| NM_005566 | 3939 | LDHA |
| NM_000221 | 3795 | KHK |
| NM_006763 | 7832 | BTG2 |
| NM_000210 | 3655 | ITGA6 |
| NM_001099294 | 85350 | KIAA1644 |
| NM_020775 | 57540 | KIAA1324 |
| NM_003931 | 8936 | WASF1 |
| NM_001024847 | 7048 | TGFBR2 |
| NM_052877 | 113000 | MED8 |
| NM_152930 | 8904 | CPNE1 |
| NM_144683 | 147000 | DHRS13 |
| NM_002292 | 3913 | LAMB2 |
| NM_001123355 | 5537 | PPP6C |
| NM_018639 | 55880 | WSB2 |
| NM_147780 | 1508 | CTSB |
| NM_004398 | 1662 | DDX10 |
| NM_032799 | 84880 | ZDHHC12 |
| NM_015456 | 25920 | COBRA1 |
| NM_201593 | 783 | CACNB2 |
| NM_016946 | 50850 | F11R |
| NR_023312 | 1153 | CIRBP |
| NM_001077619 | 137900 | UBXN2B |
| NM_012236 | 22960 | SCMH1 |
| NM_004849 | 9474 | ATG5 |
| NM_004726 | 9185 | REPS2 |
| BC013284 | 642000 | LOC642031 |
| NM_024110 | 79090 | CARD14 |
| NM_032296 | 84260 | FLYWCH1 |
| NM_001144927 | 28510 | NKIRAS2 |
| NM_207344 | 283400 | SPRYD4 |
| NM_152734 | 221500 | C6orf89 |
| NM_001142705 | 10940 | C11orf58 |
| NM_005197 | 1112 | FOXN3 |
| NM_000361 | 7056 | THBD |
| NM_032648 | 84730 | FAM167B |
| NM_002773 | 5652 | PRSS8 |
| NM_001039569 | 130300 | AP1S3 |
| NM_033215 | 89800 | PPP1R3F |
| NM_001736 | 728 | C5AR1 |
| NM_014016 | 22910 | SACM1L |
| NM_058246 | 10050 | DNAJB6 |
| NM_014369 | 26470 | PTPN18 |
| NM_015251 | 23300 | ATMIN |
| NM_001077594 | 91830 | C14orf73 |
| NM_001044385 | 65060 | ALS2CR4 |
| NM_004148 | 4814 | NINJ1 |
| NM_000859 | 3156 | HMGCR |
| NM_004781 | 9341 | VAMP3 |
| NM_001949 | 1871 | E2F3 |
| NM_001542 | 3321 | IGSF3 |
| NM_138444 | 115200 | KCTD12 |
| NM_032335 | 84300 | PHF6 |
| NM_005374 | 4355 | MPP2 |
| NM_032726 | 84810 | PLCD4 |
| NM_005566 | 3939 | LDHA |
| NM_022770 | 64780 | GINS3 |
| NM_024092 | 79070 | TMEM109 |
| NM_024033 | 79000 | C7orf49 |
| NM_198252 | 2934 | GSN |
| NM_004295 | 9618 | TRAF4 |
| NM_144765 | 10200 | MPZL2 |
| NM_001100389 | 201900 | TMEM192 |
| NM_016353 | 51200 | ZDHHC2 |
| NM_015055 | 23080 | SWAP70 |
| NM_002393 | 4194 | MDM4 |
| NM_013399 | 29960 | C16orf5 |
| NM_012293 | 7837 | PXDN |
| NM_014604 | 30850 | TAX1BP3 |
| NM_006500 | 4162 | MCAM |
| NM_134421 | 3241 | HPCAL1 |
| NM_003881 | 8839 | WISP2 |
| NM_203468 | 954 | ENTPD2 |
| NM_078483 | 206400 | SLC36A1 |
| NM_005570 | 3998 | LMAN1 |
| NM_000320 | 5860 | QDPR |
| NM_021202 | 58480 | TP53INP2 |
| NM_012455 | 23550 | PSD4 |
| NM_002775 | 5654 | HTRA1 |
| NM_014874 | 9927 | MFN2 |
| NM_001001349 | 28510 | NKIRAS2 |
| NM_014400 | 27080 | LYPD3 |
| NM_014038 | 28970 | BZW2 |
| NM_198066 | 64840 | GNPNAT1 |
| NM_032827 | 84910 | ATOH8 |
| NM_001712 | 634 | CEACAM1 |
| NM_001251 | 968 | CD68 |
| NM_001043352 | 7170 | TPM3 |
| NM_002569 | 5045 | FURIN |
| NM_000854 | 2953 | GSTT2 |
| NM_004879 | 9538 | EI24 |
| NM_002130 | 3157 | HMGCS1 |
| NM_018645 | 55500 | HES6 |
| NM_004233 | 9308 | CD83 |
| NM_001098784 | 23620 | FAM89B |
| NM_013346 | 29930 | SNX12 |
| NM_015458 | 66040 | MTMR9 |
| NM_032385 | 10830 | C5orf4 |
| NM_019001 | 54460 | XRN1 |
| NM_023018 | 65220 | NADK |
| NM_152288 | 93130 | ORAI3 |
| NM_001229 | 842 | CASP9 |
| NM_207340 | 254400 | ZDHHC24 |
| NM_020810 | 57570 | TRMT5 |
| NM_001001974 | 59340 | PLEKHA1 |
| NM_022744 | 64760 | C16orf58 |
| NM_024567 | 79620 | HMBOX1 |
| NM_020400 | 57120 | LPAR5 |
| NM_001012971 | 200200 | C20orf106 |
| NM_001135147 | 64120 | SLC39A8 |
| NM_001099668 | 25990 | HIGD1A |
| NM_004124 | 2764 | GMFB |
| NM_006401 | 10540 | ANP32B |
| NM_080916 | 1716 | DGUOK |
| NM_006817 | 10960 | ERP29 |
| NM_016145 | 51400 | C19orf56 |
| NM_005371 | 4234 | METTL1 |
| NM_005980 | 6286 | S100P |
| NM_006522 | 7475 | WNT6 |
| NM_005768 | 10160 | LPCAT3 |
| NM_002755 | 5604 | MAP2K1 |
| NM_003486 | 8140 | SLC7A5 |
| NM_000107 | 1643 | DDB2 |
| NM_014862 | 9915 | ARNT2 |
| NM_005409 | 6373 | CXCL11 |
| NM_002419 | 4296 | MAP3K11 |
| NM_006367 | 10490 | CAP1 |
| NM_016498 | 51540 | MTP18 |
| NM_016946 | 50850 | F11R |
| NM_020775 | 57540 | KIAA1324 |
| NM_005094 | 11000 | SLC27A4 |
| NM_012079 | 8694 | DGAT1 |
| NM_003500 | 8309 | ACOX2 |
| NM_004235 | 9314 | KLF4 |
| NM_015266 | 23320 | SLC9A8 |
| NM_012197 | 23640 | RABGAP1 |
| NM_006850 | 11010 | IL24 |
| NM_004263 | 10500 | SEMA4F |
| NM_020988 | 2775 | GNAO1 |
| NM_017631 | 55600 | DDX60 |
| NM_004210 | 9148 | NEURL |
| NM_015387 | 25840 | MOBKL3 |

**Appendix Supplementary Methods**

## *Cell lines and plasmids*

HeLa Kyoto cell line was originally obtained from S. Narumiya (Kyoto University, Japan) and cultured in Dulbecco's modified eagle medium (DMEM; GIBCO) supplemented with 10% (v/v) fetal bovine serum (PAA Laboratories) and 1% (v/v) penicillin–streptomycin (Invitrogen). Live-cell imaging experiments were performed using reporter cell lines that were described before ([Konno et al., 2008](#_ENREF_4); [Logarinho et al., 2012](#_ENREF_5)). Live-cell imaging was performed in complete DMEM medium without phenol red and riboflavin to reduce auto fluorescence. GFP-JAM-A fusion plasmid were produced by cloning a recombinant cassette containing WT or MUT versions (see Fig. S3) of mouse JAM-A (Geneblocks, IDT) into the XhoI/EcoRI sites of the pEFGP-C1 plasmid. JAM-AMUT fusion contains point mutations in all the bases that pair with miR-34/449 family at the two predicted miR-34/449 binding sites, without disturbing JAM-A open reading frame. To generate a HeLa stable cell line overexpressing JAM-A from a constitutive promoter we generated a lentiviral vector expressing JAM-A from a Human elongation factor-1 alpha (EF1a) promoter. Recombinant cassettes containing a WT version of mouse JAM-A or S. piogenes Cas9 were inserted in the EcoRI site of the lentiviral vector, which also contained a blasticidin cassette after IRES element. HeLa Kyoto cell line expressing a centrosome marker (centrin-2-EGFP) and a microtubule marker (α-tubulin fused to monomeric red fluorescent protein, α-tubulin-mRFP) ([Logarinho et al., 2012](#_ENREF_5)) was infected with these lentiviruses separately and a pool of clones was selected using blasticidin (6 µg/ml) for each infection. This pools of clones were expanded and used for the in vitro rescue experiments.

*Transfection of miRNA mimics, siRNAs, and plasmids*

The cortical embryonic cortical miRNA library was constructed based on the cortical miRNA expression data from Yao et al. (Table S1) ([Yao et al., 2012](#_ENREF_6)). The miRNA mimics representing cortical embryonic miRNAs were derived from the miScript miRNA mimic library V13.0. miRNA mimics were transfected in liquid phase with either HiPerfect (Qiagen) for the screening and differential gene expression experiments or Lipofectamine RNAIMax (Invitrogen) for the *in vitro* experiments (Figures 1D-E, 4), following the manufacturer's instructions. Final miRNA mimic and siRNA concentrations were 10 nM in all conditions. For the screening, cells were seeded into wells of a 96-well microtitre plates containing miRNA mimics and transfection mix and incubated for approximately 48 h before imaging. For the cell biology microscopy assays (Figure 1A-B) 8-well labtek plates were used. For the JAM-A direct targeting assay (Figure S3), 20 ng of GFP-JAM-A fusion plasmids were co-transfected with 20 ng eGFP expression plasmids and miRNA mimic to a final concentration of 20 nM in 8-well Labteks, and incubated for 36 h before lysing the cells for protein blotting assays. All reagents were used according to the manufacturer’s instructions.

*Mouse genotyping*

For genotyping, the oligomeres and PCR conditions described before were used ([Bao et al., 2012](#_ENREF_1); [Concepcion et al., 2012](#_ENREF_2)).

### *Microarray Expression analysis*

HeLa Kyoto cells were transfected in triplicate with final 10 nM miR-449 mimics or negative control mimic and harvested 48 h. later. RNA from cells was prepared using RNAeasy kit (QIAGEN). 1 µg of total RNA from each triplicate (six samples in total) was sent to Functional Genomics Center Zurich (FGCZ) for sample processing and hybridization to Human Gene Expression v2 4x44K V2 Agilent microarrays. FGCZ performed the quality control and data normalization. Microarray data analysis was carried out using the R language with Bioconductor packages ([Gentleman et al., 2004](#_ENREF_3)). Microarray data were deposited in the Gene Expression Omnibus (GEO) database (Accession Code: GSE85735).

*Real-Time qPCR*

To quantify the abundance of mRNAs, total RNA was isolated from heterozygous controls and miR-34/49 KO E14 cerebral cortices, or HeLa Kyoto WT cells transfected with the corresponding miRNA mimic or siRNA using TRIzol reagent (Invitrogen). cDNA was synthesized from 500 ng of total RNA using Superscript II (Invitrogen) with random hexamer primers. Real-time PCR was performed on a C1000 Thermal Cycler (Bio-Rad). Quantification was performed using CFX Manager software (Bio-Rad) with data normalized to the level of PGK1 mRNA for mouse cortices, and to GAPDH for HeLa samples, respectively.

miRNA and U6 levels in micro-dissected ventricular zone samples from E14 mouse embryonic cortices were determined by using Taqman microRNA Assays (Applied Biosystems) after total RNA isolation using TRIzol reagent (Invitrogen). Quantification of the absolute miRNA copy number in the measured samples was performed by using standard curves of PAGE purified 5’-phosphorylated oligomeres corresponding to the reference sequences for each measured miRNA. Absolute copy numbers of miRNAs were calculated by interpolation of the Ct values of individual samples into the corresponding standard curves. Mature miRNAs were normalized to U6 RNA levels. Standard curve for U6 RNA was derived from serial dilutions of total U6 RNA, enabling the calculation of relative miRNA levels.

*Cell-Cycle duration and cell-cycle exit in vivo assays*

For the cell cycle exit assay (Figure S2C-E), BrdU labeling was carried out by intraperitoneal injection of 100 μl solution containing 10 mg/ml BrdU (Sigma) in PBS into pregnant mice carrying E15 embryos. Twenty-four h later, mice were killed and embryonic brains were dissected, fixed, and sectioned as described before. Sections were stained with anti-Ki-67 antibody (1:100, BD pharmingen) and anti-BrdU antibody (1:100, clone BU1/75, Abcam). For the cell cycle duration assay (Figure S2F-I), BrdU labeling was carried out by intraperitoneal injection of 100 μl solution containing 10 mg/ml EdU (Sigma) in PBS into pregnant mice carrying E15 embryos. After 2.5 h, BrdU labeling was carried out by intraperitoneal injection of 100 μl solution containing 10 mg/ml BrdU (Sigma) in PBS. 30 min later, mice were killed and embryonic brains were dissected, fixed and sectioned as described above. EdU was detected with the Click-iT EdU Alexa Fluor 488 Imaging kit (Thermo Fisher Scientific). Brain sections were also stained with anti-BrdU antibody (1:100, clone BU1/75, Abcam).

*Mouse genotyping primers*

| Name | Sequence | PCR product |
| --- | --- | --- |
| miR-449-F | GATTCTCACAACTGATGTAG | miR-449abc wild-type band: 368 bp  miR-449abc KO band: 217 bp |
| miR-449-R1 | ACAATGGTTAGTACTTTCAC |  |
| miR-449-R2 | ATGCACAGATATAAGTGCAG |  |
| AV209 | ATACCCTGGATCTCCAACAG | miR-34b~c wild-type band: 377 bp  miR-34b~c KO band: 249 bp |
| AV208 | ACAAGACCCTCACCTGAATG |  |
| AV232 | GCCATCCTGTTGAGGGACTA |  |

*Primers used in real-time PCR*

| Name | Sequence |
| --- | --- |
| mouse Pgk1 forward | AACCTCCGCTTTCATGTAGAG |
| mouse Pgk1 reverse | GACATCTCCTAGTTTGGACAGTG |
| mouse Pmf1 forward | GAAACTCCTGGACGCCATAG |
| mouse Pmf1 reverse | GCAACTGAGCCACAAACTTG |
| mouse Nup210 forward | ATCTCATCCACGGCATTCAG |
| mouse Nup210 reverse | AATTGTCCAGTCGAAGACCAG |
| mouse Rabgap1 forward | CATCGACCTGCTCTTATGTGAG |
| mouse Rabgap1 reverse | CTGAGCGGTATCTTTTAGGGAG |
| mouse JAM-A forward | CTCTTCACGTCTATGATCCTGG |
| mouse JAM-A reverse | AGGAGAAGCCAGAGTAGGTG |
| mouse NOTCH1 forward | ATGTCAATGTTCGAGGACCAG |
| mouse NOTCH1 reverse | TCACTGTTGCCTGTCTCAAG |
| mouse Dll1 forward | ATTCCCCTTCGGCTTCAC |
| mouse Dll1 reverse | CTTTCTGGGTTTTCTGTTGCG |
| human JAM-A forward | CAGATGCCAAGAAAACCCG |
| human JAM-A reverse | GGCTGTCCCATATCCATTCTG |
| human GAPDH forward | ACATCGCTCAGACACCATG |
| human GAPDH reverse | TGTAGTTGAGGTCAATGAAGGG |

*miRNA reference sequence oligos*

| miRNA | Real-Time PCR Reference Sequence |
| --- | --- |
| mmu-miR-34a-5p | UGGCAGUGUCUUAGCUGGUUGU |
| mmu-miR-34b-5p | AGGCAGUGUAAUUAGCUGAUUGU |
| mmu-miR-34c-5p | AGGCAGUGUAGUUAGCUGAUUGC |
| mmu-miR-449a | UGGCAGUGUAUUGUUAGCUGGU |
| mmu-miR-449b | AGGCAGUGUUGUUAGCUGGC |
| mmu-miR-449c-5p | AGGCAGUGCAUUGCUAGCUGGCUGU |
| mmu-miR-7a-1 | UGGAAGACUAGUGAUUUUGUUGU |

*miRNA mimic/siRNA reagents*

| Name | Strand | Sequence |
| --- | --- | --- |
| Non-targeting siRNA control  /miRNA mimic negative control | sense | UACGACCGGUCUAUCGUAGtt |
| siRNA targeting JAM-A | sense | CCAUCCAAGCCUACAGUUAtt |

*Embryonic cortical miRNA mimic library*

| miRNA mimic name | Strand | Sequence |
| --- | --- | --- |
| hsa-let-7a | guide | AACUAUACAACCUACUACCUCA |
| hsa-let-7b | guide | AACCACACAACCUACUACCUCA |
| hsa-let-7c | guide | AACCAUACAACCUACUACCUCA |
| hsa-let-7d | guide | AACUAUGCAACCUACUACCUCU |
| hsa-let-7e | guide | AACUAUACAACCUCCUACCUCA |
| hsa-let-7f | guide | AACUAUACAAUCUACUACCUCA |
| hsa-let-7g | guide | AACUGUACAAACUACUACCUCA |
| hsa-let-7i | guide | AACAGCACAAACUACUACCUCA |
| hsa-miR-1 | guide | AUACAUACUUCUUUACAUUCCA |
| hsa-miR-100 | guide | CACAAGUUCGGAUCUACGGGUU |
| hsa-miR-101 | guide | UUCAGUUAUCACAGUACUGUA |
| hsa-miR-106a | guide | CUACCUGCACUGUAAGCACUUUU |
| hsa-miR-106a* | guide | GUAAGAAGUGCUUACAUUGCAG |
| hsa-miR-106b | guide | AUCUGCACUGUCAGCACUUUA |
| hsa-miR-106b* | guide | GCAGCAAGUACCCACAGUGCGG |
| hsa-miR-107 | guide | UGAUAGCCCUGUACAAUGCUGCU |
| hsa-miR-124 | guide | GGCAUUCACCGCGUGCCUUA |
| hsa-miR-125a-3p | guide | GGCUCCCAAGAACCUCACCUGU |
| hsa-miR-125a-5p | guide | UCACAGGUUAAAGGGUCUCAGGGA |
| hsa-miR-125b | guide | UCACAAGUUAGGGUCUCAGGGA |
| hsa-miR-125b-1* | guide | AGCUCCCAAGAGCCUAACCCGU |
| hsa-miR-125b-2* | guide | GUCCCAAGAGCCUGACUUGUGA |
| hsa-miR-127-3p | guide | AGCCAAGCUCAGACGGAUCCGA |
| hsa-miR-128 | guide | AAAGAGACCGGUUCACUGUGA |
| hsa-miR-130a | guide | AUGCCCUUUUAACAUUGCACUG |
| hsa-miR-130b | guide | AUGCCCUUUCAUCAUUGCACUG |
| hsa-miR-134 | guide | CCCCUCUGGUCAACCAGUCACA |
| hsa-miR-135a | guide | UCACAUAGGAAUAAAAAGCCAUA |
| hsa-miR-135a* | guide | CGCCACGGCUCCAAUCCCUAUA |
| hsa-miR-135b* | guide | CCCAUGGCUUUUAGCCCUACAU |
| hsa-miR-140-3p | guide | CCGUGGUUCUACCCUGUGGUA |
| hsa-miR-143 | guide | GAGCUACAGUGCUUCAUCUCA |
| hsa-miR-146a | guide | AACCCAUGGAAUUCAGUUCUCA |
| hsa-miR-146b-5p | guide | AGCCUAUGGAAUUCAGUUCUCA |
| hsa-miR-151-3p | guide | CCUCAAGGAGCUUCAGUCUAG |
| hsa-miR-151-5p | guide | ACUAGACUGUGAGCUCCUCGA |
| hsa-miR-152 | guide | CCAAGUUCUGUCAUGCACUGA |
| hsa-miR-15a | guide | CACAAACCAUUAUGUGCUGCUA |
| hsa-miR-15b* | guide | UAGAGCAGCAAAUAAUGAUUCG |
| hsa-miR-17 | guide | CUACCUGCACUGUAAGCACUUUG |
| hsa-miR-181a | guide | ACUCACCGACAGCGUUGAAUGUU |
| hsa-miR-181b | guide | ACCCACCGACAGCAAUGAAUGUU |
| hsa-miR-181c | guide | ACUCACCGACAGGUUGAAUGUU |
| hsa-miR-181d | guide | ACCCACCGACAACAAUGAAUGUU |
| hsa-miR-185 | guide | UCAGGAACUGCCUUUCUCUCCA |
| hsa-miR-186 | guide | AGCCCAAAAGGAGAAUUCUUUG |
| hsa-miR-191 | guide | CAGCUGCUUUUGGGAUUCCGUUG |
| hsa-miR-192 | guide | GGCUGUCAAUUCAUAGGUCAG |
| hsa-miR-199a-3p // hsa-miR-199b-3p | guide | UAACCAAUGUGCAGACUACUGU |
| hsa-miR-206 | guide | CCACACACUUCCUUACAUUCCA |
| hsa-miR-20a | guide | CUACCUGCACUAUAAGCACUUUA |
| hsa-miR-20b | guide | CUACCUGCACUAUGAGCACUUUG |
| hsa-miR-21 | guide | UCAACAUCAGUCUGAUAAGCUA |
| hsa-miR-219-1-3p | guide | CGGGACGUCCAGACUCAACUCU |
| hsa-miR-219-2-3p | guide | ACAGAUGUCCAGCCACAAUUCU |
| hsa-miR-219-5p | guide | AGAAUUGCGUUUGGACAAUCA |
| hsa-miR-221 | guide | GAAACCCAGCAGACAAUGUAGCU |
| hsa-miR-23a | guide | GGAAAUCCCUGGCAAUGUGAU |
| hsa-miR-23b | guide | GGUAAUCCCUGGCAAUGUGAU |
| hsa-miR-24 | guide | CUGUUCCUGCUGAACUGAGCCA |
| hsa-miR-25 | guide | UCAGACCGAGACAAGUGCAAUG |
| hsa-miR-25* | guide | CAAUUGCCCAAGUCUCCGCCU |
| hsa-miR-26a | guide | AGCCUAUCCUGGAUUACUUGAA |
| hsa-miR-26b | guide | ACCUAUCCUGAAUUACUUGAA |
| hsa-miR-27a | guide | GCGGAACUUAGCCACUGUGAA |
| hsa-miR-27b | guide | GCAGAACUUAGCCACUGUGAA |
| hsa-miR-296-3p | guide | GGAGAGCCUCCACCCAACCCUC |
| hsa-miR-298 | guide | UGGGAGAACCUCCCUGCUUCUGCU |
| hsa-miR-29a | guide | UAACCGAUUUCAGAUGGUGCUA |
| hsa-miR-29b | guide | AACACUGAUUUCAAAUGGUGCUA |
| hsa-miR-29c | guide | UAACCGAUUUCAAAUGGUGCUA |
| hsa-miR-301b | guide | GCUUUGACAAUAUCAUUGCACUG |
| hsa-miR-302c* | guide | CAGCAGGUACCCCCAUGUUAAA |
| hsa-miR-302d | guide | ACACUCAAACAUGGAAGCACUUA |
| hsa-miR-302d* | guide | GCAAGUGCCUCCAUGUUAAAGU |
| hsa-miR-302e | guide | AAGCAUGGAAGCACUUA |
| hsa-miR-302f | guide | AAACAUGGAAGCAAUUA |
| hsa-miR-30a | guide | CUUCCAGUCGAGGAUGUUUACA |
| hsa-miR-30a* | guide | GCUGCAAACAUCCGACUGAAAG |
| hsa-miR-30b | guide | AGCUGAGUGUAGGAUGUUUACA |
| hsa-miR-30b* | guide | GAAGUAAACAUCCACCUCCCAG |
| hsa-miR-30c | guide | GCUGAGAGUGUAGGAUGUUUACA |
| hsa-miR-30c-1* | guide | GGAGUAAACAACCCUCUCCCAG |
| hsa-miR-30c-2* | guide | AGAGUAAACAGCCUUCUCCCAG |
| hsa-miR-30d | guide | CUUCCAGUCGGGGAUGUUUACA |
| hsa-miR-30d* | guide | GCAGCAAACAUCUGACUGAAAG |
| hsa-miR-30e | guide | CUUCCAGUCAAGGAUGUUUACA |
| hsa-miR-30e* | guide | GCUGUAAACAUCCGACUGAAAG |
| hsa-miR-320a | guide | UCGCCCUCUCAACCCAGCUUUU |
| hsa-miR-320b | guide | UUGCCCUCUCAACCCAGCUUUU |
| hsa-miR-320c | guide | ACCCUCUCAACCCAGCUUUU |
| hsa-miR-320d | guide | UCCUCUCAACCCAGCUUUU |
| hsa-miR-323-3p | guide | AGAGGUCGACCGUGUAAUGUG |
| hsa-miR-331-3p | guide | UUCUAGGAUAGGCCCAGGGGC |
| hsa-miR-335 | guide | ACAUUUUUCGUUAUUGCUCUUGA |
| hsa-miR-33a | guide | UGCAAUGCAACUACAAUGCAC |
| hsa-miR-33b | guide | GCAAUGCAACAGCAAUGCAC |
| hsa-miR-340 | guide | AAUCAGUCUCAUUGCUUUAUAA |
| hsa-miR-342-3p | guide | ACGGGUGCGAUUUCUGUGUGAGA |
| hsa-miR-345 | guide | GAGCCCUGGACUAGGAGUCAGC |
| hsa-miR-34a | guide | ACAACCAGCUAAGACACUGCCA |
| hsa-miR-34b | guide | AUGGCAGUGGAGUUAGUGAUUG |
| hsa-miR-34c-5p | guide | GCAAUCAGCUAACUACACUGCCU |
| hsa-miR-363 | guide | UACAGAUGGAUACCGUGCAAUU |
| hsa-miR-369-5p | guide | GCGAAUAUAACACGGUCGAUCU |
| hsa-miR-374a | guide | CACUUAUCAGGUUGUAUUAUAA |
| hsa-miR-374b | guide | CACUUAGCAGGUUGUAUUAUAU |
| hsa-miR-378 | guide | CCUUCUGACUCCAAGUCCAGU |
| hsa-miR-379 | guide | CCUACGUUCCAUAGUCUACCA |
| hsa-miR-382 | guide | CGAAUCCACCACGAACAACUUC |
| hsa-miR-409-3p | guide | AGGGGUUCACCGAGCAACAUUC |
| hsa-miR-423-3p | guide | ACUGAGGGGCCUCAGACCGAGCU |
| hsa-miR-433 | guide | ACACCGAGGAGCCCAUCAUGAU |
| hsa-miR-448 | guide | AUGGGACAUCCUACAUAUGCAA |
| hsa-miR-449a | guide | ACCAGCUAACAAUACACUGCCA |
| hsa-miR-449b | guide | GCCAGCUAACAAUACACUGCCU |
| hsa-miR-451 | guide | AACUCAGUAAUGGUAACGGUUU |
| hsa-miR-485-5p | guide | GAAUUCAUCACGGCCAGCCUCU |
| hsa-miR-487a | guide | AACUGGAUGUCCCUGUAUGAUU |
| hsa-miR-487b | guide | AAGUGGAUGACCCUGUACGAUU |
| hsa-miR-494 | guide | GAGGUUUCCCGUGUAUGUUUCA |
| hsa-miR-495 | guide | AAGAAGUGCACCAUGUUUGUUU |
| hsa-miR-499-5p | guide | AAACAUCACUGCAAGUCUUAA |
| hsa-miR-503 | guide | CUGCAGAACUGUUCCCGCUGCUA |
| hsa-miR-532-5p | guide | ACGGUCCUACACUCAAGGCAUG |
| hsa-miR-541 | guide | AGUCCAGAUUCUGUGCCCACCA |
| hsa-miR-542-3p | guide | UUUCAGUUAUCAAUCUGUCACA |
| hsa-miR-543 | guide | AAGAAGUGCACCGCGAAUGUUU |
| hsa-miR-598 | guide | UGACGAUGACAACGAUGACGUA |
| hsa-miR-7 | guide | ACAACAAAAUCACUAGUCUUCCA |
| hsa-miR-708 | guide | CCCAGCUAGAUUGUAAGCUCCUU |
| hsa-miR-9 | guide | UCAUACAGCUAGAUAACCAAAGA |
| hsa-miR-9* | guide | ACUUUCGGUUAUCUAGCUUUAU |
| hsa-miR-92a | guide | ACAGGCCGGGACAAGUGCAAUA |
| hsa-miR-92b | guide | GGAGGCCGGGACGAGUGCAAUA |
| hsa-miR-93 | guide | CUACCUGCACGAACAGCACUUUG |
| hsa-miR-98 | guide | AACAAUACAACUUACUACCUCA |
| hsa-miR-99a | guide | CACAAGAUCGGAUCUACGGGUU |
| hsa-miR-99a* | guide | CAGACCCAUAGAAGCGAGCUUG |
| hsa-miR-99b | guide | CGCAAGGUCGGUUCUACGGGUG |
| hsa-miR-99b* | guide | CGGACCCACAGACACGAGCUUG |

Note:* represents miRNA product from the 3’arm of the hairpin

**Appendix References**

Bao, J., Li, D., Wang, L., Wu, J., Hu, Y., Wang, Z., Chen, Y., Cao, X., Jiang, C., Yan, W.*, et al.* (2012). MicroRNA-449 and microRNA-34b/c function redundantly in murine testes by targeting E2F transcription factor-retinoblastoma protein (E2F-pRb) pathway. The Journal of biological chemistry *287*, 21686-21698.

Concepcion, C.P., Han, Y.C., Mu, P., Bonetti, C., Yao, E., D'Andrea, A., Vidigal, J.A., Maughan, W.P., Ogrodowski, P., and Ventura, A. (2012). Intact p53-dependent responses in miR-34-deficient mice. PLoS genetics *8*, e1002797.

Gentleman, R.C., Carey, V.J., Bates, D.M., Bolstad, B., Dettling, M., Dudoit, S., Ellis, B., Gautier, L., Ge, Y., Gentry, J.*, et al.* (2004). Bioconductor: open software development for computational biology and bioinformatics. Genome biology *5*, R80.

Konno, D., Shioi, G., Shitamukai, A., Mori, A., Kiyonari, H., Miyata, T., and Matsuzaki, F. (2008). Neuroepithelial progenitors undergo LGN-dependent planar divisions to maintain self-renewability during mammalian neurogenesis. Nature cell biology *10*, 93-101.

Logarinho, E., Maffini, S., Barisic, M., Marques, A., Toso, A., Meraldi, P., and Maiato, H. (2012). CLASPs prevent irreversible multipolarity by ensuring spindle-pole resistance to traction forces during chromosome alignment. Nature cell biology *14*, 295-303.

Yao, M.J., Chen, G., Zhao, P.P., Lu, M.H., Jian, J., Liu, M.F., and Yuan, X.B. (2012). Transcriptome analysis of microRNAs in developing cerebral cortex of rat. BMC genomics *13*, 232.
