# Supplementary material for: MicroRNA‐34/449 controls mitotic spindle orientation during mammalian cortex development
Source: EMBO J. 2016 Oct 5;35(22):2386–98. doi: 10.15252/embj.201694056 (PMC5109238; doi:10.15252/embj.201694056)
Supplement: Supplementary file 2 — Expanded View Figures PDF [file EMBJ-35-2386-s002.pdf]

Expanded View Figures

A

Locus 1. mmu-miR-34a uggcagugucuuagcugguugu

Locus 2. mmu-miR-34b aggcaguguaauuagcugauugu
mmu-miR-34c aggcaguguaguuagcugauugc

Locus 3. mmu-miR-449a uggcaguguauuguuagcuggu
mmu-miR-449b aggcaguguuguuagcuggc
mmu-miR-449c aggcagugcauugcuagcugg

B

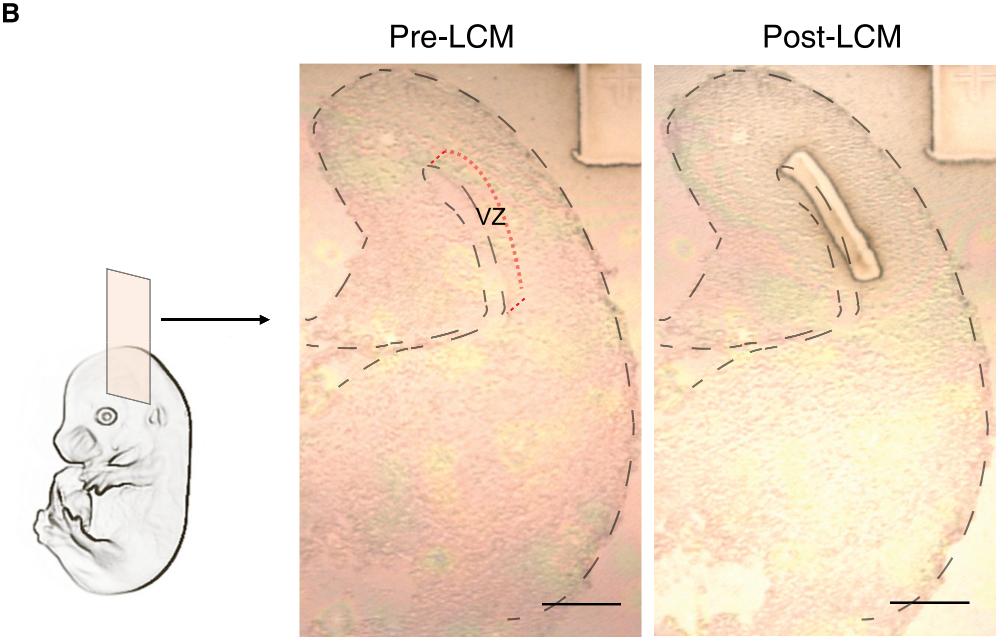

Figure EV1. miR-34/449 family locus structure and laser capture microdissection procedure.

A Sequence alignments of mature mouse miR-34/449 miRNAs. Blue letters indicate seed sequences.

B Laser capture microdissection (LCM) of the ventricular zone (VZ) of mouse cortex at embryonic day E14. Representative images of pre- and post-microdissection. Scale bar, 300 µm.

 © 2016 The Authors

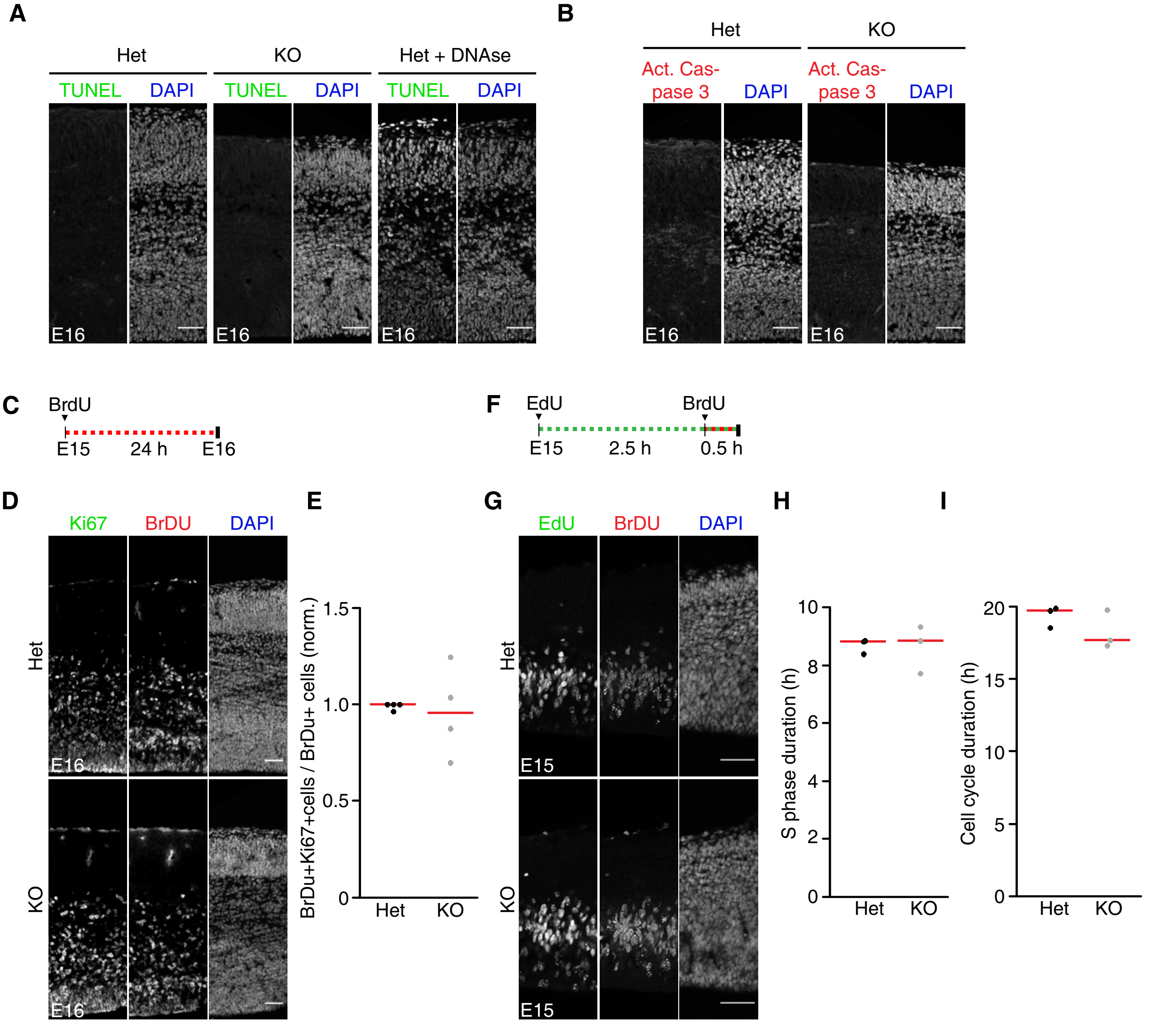

Figure EV2. Apoptosis and cell cycle phenotypes in miR-34/449 KO mouse neocortices.

A, B Confocal images of coronal sections from E16 brains of miR-34/449 KO mice and littermate controls (Het), stained with TUNEL (green) or activated caspase-3 (red) and DAPI to label all cell nuclei (blue). Cells stained for TUNEL and activated caspase-3 are not detectable in mutant and control brains. Sections were counterstained with DAPI (blue). Scale bar, 50 μm.

C Scheme of BrdU incorporation protocol in female pregnant mice to measure cell cycle exit rates of neural progenitors in the embryonic cortex.

D Confocal images of coronal sections from miR-34/449 KO mice brain compared with littermate controls (Het) in E16 embryos labeled for BrdU (24 h, red) and stained for Ki67 (green). Scale bar, 50 μm.

E Mitotic exit rates in miR-34/449 KO mice brain compared with littermate controls (Het) at E16. Cells exiting cell cycle during the BrdU incorporation (24 h) are BrdU positive but Ki67 negative. BrdU labeling and Ki67 labeling show no difference between KO embryos compared to littermate controls (n = 4 brains for each genotype group, 2 independent litters). Data were normalized (norm.) to the ventricular zone surface analyzed (100 μm) and relativized to the heterozygous control average value. P-value = 0.8294 (Het vs. KO); red bar indicates median. Significance was tested by Welch's t-test.

F Scheme of BrdU and EdU incorporation protocol in female pregnant mice to measure cell cycle duration and S phase duration in neural progenitors in the embryonic cortex.

G Confocal images of coronal sections from miR-34/449 KO mice brain compared with littermate controls (Het) in E15 embryos labeled for EdU (3 h, green) and BrdU (0.5 h, red). Scale bar, 50 μm.

H, I S phase duration and cell cycle duration in miR-34/449 KO mice brain compared with littermate controls (Het) at E15. S phase duration and cell cycle duration were calculated as described in Materials and Methods. BrdU labeling and EdU labeling show no difference between KO embryos compared to littermate controls (n = 3 brains for each genotype group, 2 independent litters); P-value = 0.9324 (S phase duration, Het vs. KO), P-value = 0.9324 (cell cycle duration, Het vs. KO); red bar indicates median. Significance was tested by Welch's t-test.

Source data are available online for this figure.

© 2016 The Authors

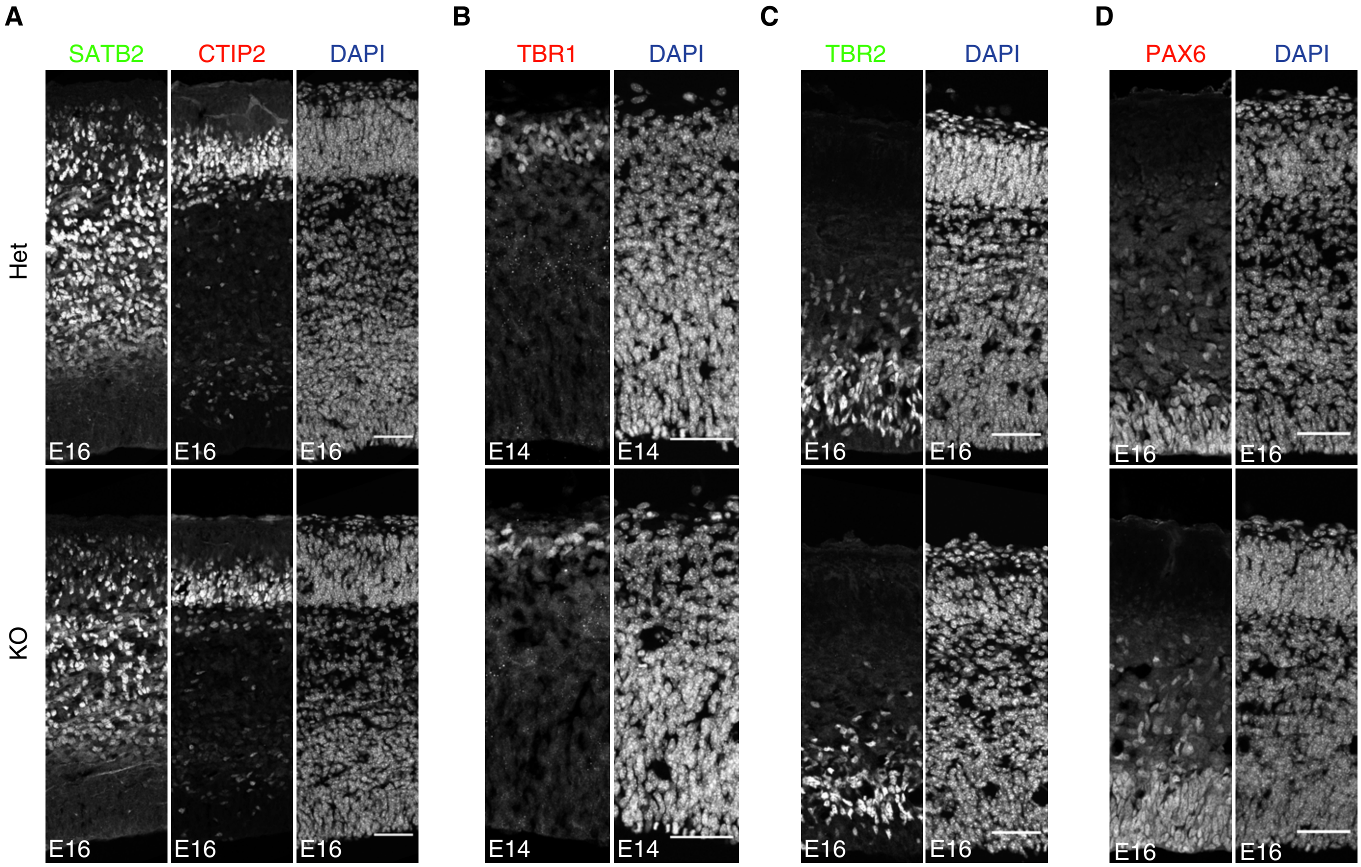

Figure EV3. miR-34/449 family is required for timely cortical neurogenesis.

A Confocal single channel images of coronal sections from E16 brains of miR-34/449 KO mice and littermate controls (Het), stained with anti-Satb2 antibody to label neurons of layers II–IV (green), anti-Ctip2 antibody to label V neurons (n = 4 brains per genotype group, 2 independent litters), and DAPI to label cell nuclei (blue). Scale bars: 50 μm.

B Confocal single channel images of coronal sections from E14 brains of miR-34/449 KO mice and littermate controls (Het), stained with anti-Tbr1 antibody to label layer IV neurons (n = 3 brains per genotype group, 2 independent litters) and DAPI to label cell nuclei (blue). Scale bars: 50 μm.

C Confocal single channel images of coronal sections from E16 brains of miR-34/449 KO mice and littermate controls (Het), stained with anti-Tbr2 antibody to label intermediate progenitors (n = 5 brains for each genotype group, 3 independent litters) and DAPI to label cell nuclei (blue). Scale bars: 50 μm.

D Confocal single channel images of coronal sections from E16 brains of miR-34/449 KO mice and littermate controls (Het), stained with anti-Pax6 antibody to label radial glia progenitors (n = 7 brains per genotype group, 5 independent litters) and DAPI to label cell nuclei (blue). Scale bars: 50 μm.

 © 2016 The Authors

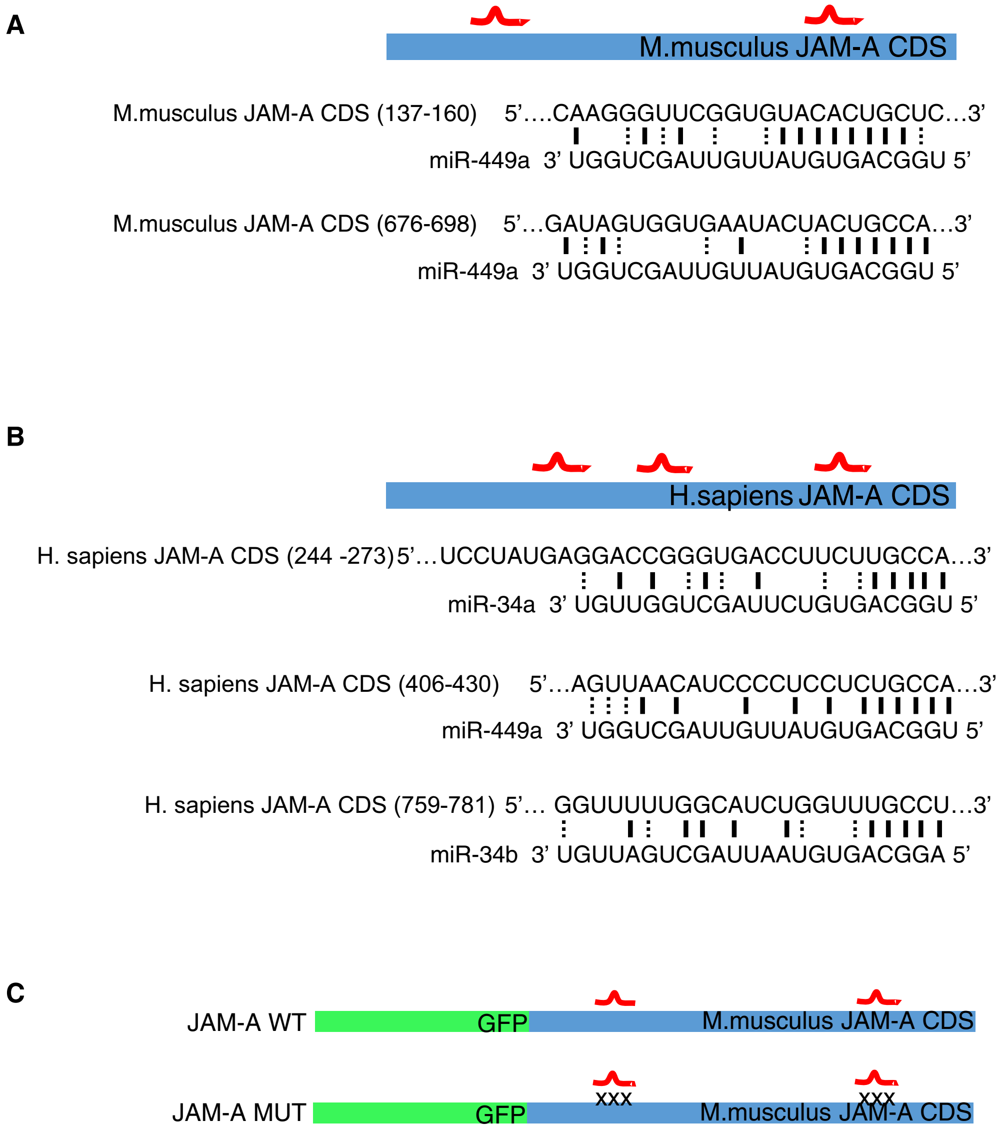

Figure EV4. miR-34/449 binding sites on mouse and human JAM-A.

A, B A schematic representation of the predicted miR-34/449 binding sites in the mouse and human JAM-A CDS, respectively.

C Schematic representation of the GFP–JAM-A fusion reporters containing WT (WT) or mutant (MUT) JAM-A CDS for the binding of miR-34/449 miRNA family.

© 2016 The Authors

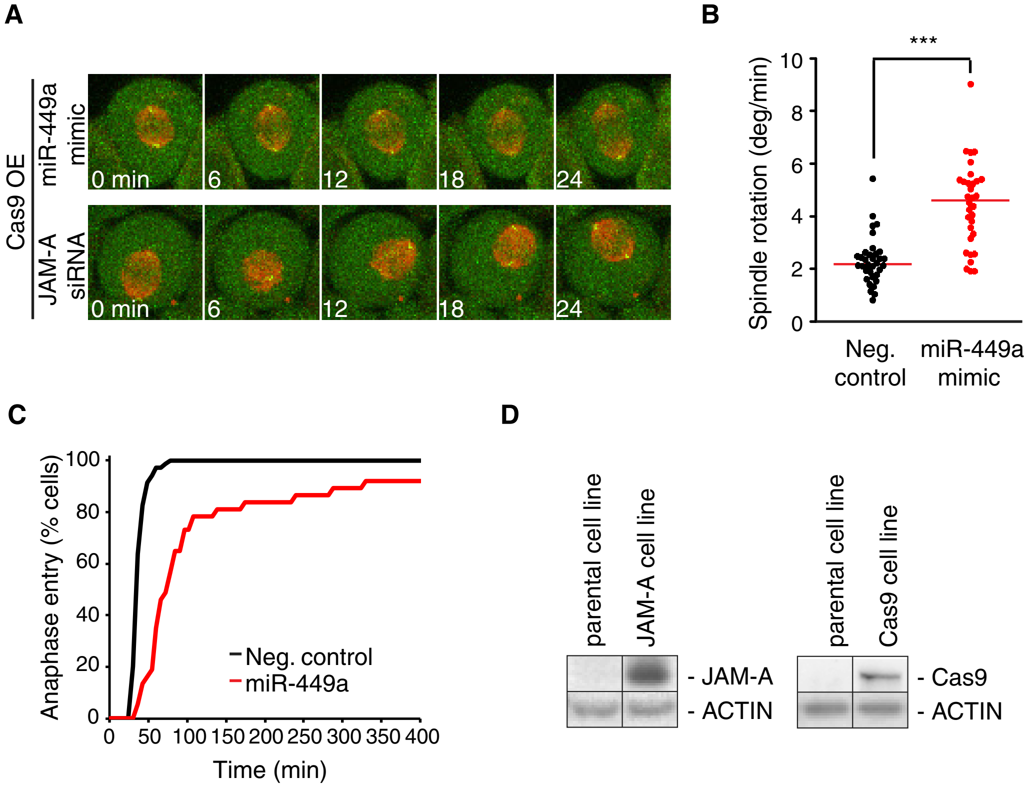

Figure EV5. JAM-A overexpression rescue of miR-449-induced spindle defects is specific.

A Confocal time-lapse images of metaphase HeLa cells stably expressing a centriole marker (centrin-2–eGFP; green), a microtubule marker (α-tubulin–mRFP; red), and Cas9, 48 h after transfection of miR-449a mimic, JAM-A siRNA, or nontargeting negative control siRNA. Scale bar, 10 μm.

B Quantification of spindle rotation for cells as shown in (A). Each dot represents a single cell. Red bar indicates median. Significance was tested by pairwise *t*-test with Bonferroni correction. ****P*-value = 7.9e-12 (Neg. control vs. miR449 mimic, under JAM-A overexpression).

C Cumulative histogram of mitotic duration from nuclear envelope breakdown until anaphase onset, measured for cells as shown in (A); $n \geq 20$ cells for all conditions, 2 independent replicates.

D Whole-cell extracts from the HeLa Kyoto stable cell lines used in Figs 5 and EV5 expressing JAM-A and Cas9 were subjected to immunoblot analysis to detect mouse JAM-A, Cas9, and actin as an endogenous control protein.

Source data are available online for this figure.

 © 2016 The Authors
